# Toxicity, Dose Intensity, and Clinical Outcomes with First-Line Enfortumab Vedotin Plus Pembrolizumab in Advanced Urothelial Carcinoma: A Multicenter Real-World Study

**DOI:** 10.3390/curroncol33090542

**Published:** 2026-09-09

**Authors:** Samad Sayed, Rishikesh Kumar, Martin Zarba, Md Mahsin, Amina Taleb, Lateefah Alshammari, Simon Mairs, Naveen Basappa, Michael Kolinsky, Meghan Mahoney, Vishal Navani, Tina Cheng, Safiya Karim, Richard Lee-Ying, Steven Yip, Daniel Y. C. Heng, Scott North, Nimira Alimohamed

**Affiliations:** 1 Arthur J.E. Child Comprehensive Cancer Centre, University of Calgary, Calgary, AB T2N5G2, Canada; 2 Cross Cancer Institute, University of Alberta, Edmonton, AB T6G1Z2, Canada; 3 Jack Ady Cancer Centre, Alberta Health Services, Lethbridge, AB T1J1W5, Canada; 4 Alberta Health Services, Red Deer, AB T4N4E7, Canada

**Keywords:** urothelial carcinoma, enfortumab vedotin, pembrolizumab, real-world evidence, toxicity, dose modification, biomarker

## Abstract

Background: Enfortumab vedotin plus pembrolizumab (EVP) is the standard first-line treatment for locally advanced or metastatic urothelial carcinoma (la/mUC); however, real-world toxicity patterns, timing of onset, and prognostic significance of treatment-related adverse events (AEs) remain incompletely characterized. Methods: We conducted a retrospective, multicenter analysis of 60 la/mUC patients treated with first-line EVP in Alberta, Canada (September 2024–January 2026). Treatment related toxicities, time to AE onset, dose modifications, and treatment discontinuation were assessed. Progression-free survival (PFS) and overall survival (OS) were analyzed. Results: The median age was 69 years, 82% of patients were male, and 80% had metastatic disease at treatment initiation. Histology was pure urothelial in 82% and mixed in 18%. Median follow-up was 10.3 months, and the median number of EV and pembrolizumab cycles was seven and eight, respectively. Rash (63%), fatigue (57%), and peripheral neuropathy (47%) were the most common adverse events, with median onset at 17, 29, and 90 days, respectively. Dose reductions occurred in 62%, dose delays in 43%, and treatment discontinuation in 38%. An initial enfortumab vedotin dose of 1.25 mg/kg was associated with improved PFS (HR 0.30, 95% CI 0.13–0.68; *p* = 0.004) and OS (HR 0.32, 95% CI 0.10–0.97; *p* = 0.044) compared with 1.0 mg/kg. Neuropathy was associated with improved PFS (HR 0.32, 95% CI 0.14–0.73; *p* = 0.007) and OS (HR 0.24, 95% CI 0.07–0.87; *p* = 0.029), while rash was associated with improved OS (HR 0.31, 95% CI 0.11–0.91; *p* = 0.032). Conclusions: EVP was associated with frequent treatment-related adverse events requiring dose modifications, and the development of rash and peripheral neuropathy was associated with favorable survival outcomes.

## Simple Summary

The standard first-line treatment for locally advanced and metastatic urothelial carcinoma is enfortumab vedotin plus pembrolizumab. Patients treated in routine clinical practice often differ from those enrolled in clinical trials, making real-world safety data very important. In this multicenter study, we evaluated the safety, efficacy and treatment modifications associated with this regimen among patients treated across Alberta, Canada. We noted that rash, fatigue, and neuropathy were the most common adverse events, and treatment modifications such as dose delays, dose reductions, and treatment discontinuation were frequently required. In addition, patients who developed rash or peripheral neuropathy experienced more favorable survival outcomes, although these associations require confirmation in larger studies. These findings provide real-world evidence to help clinicians anticipate and manage treatment-related toxicities, support patient counseling, and identify potential clinical markers associated with treatment outcomes that warrant further investigation.

## 1. Background

Bladder cancer remains a major cause of cancer morbidity and mortality worldwide, with an estimated 635,000 new diagnoses and 228,000 deaths in 2024 [1]. The first-line treatment for patients with locally advanced or metastatic urothelial carcinoma (la/mUC) was platinum-based chemotherapy for many years; however, outcomes remained limited, with median overall survival generally ranging around 12–15 months [2,3]. In recent years, first-line treatment has shifted with the introduction of enfortumab vedotin (EV), a nectin-4 directed antibody–drug conjugate that delivers the monomethyl auristatin E (microtubule disrupting agent), and pembrolizumab, a programmed death-1 (PD-1) immune checkpoint inhibitor [4,5]. Their combination is supported by a strong biological rationale, whereby ADC-mediated cytotoxicity and immunogenic cell death may enhance antitumor immune responses through PD-1 blockade [6].

The phase III EV-302 trial established the clinical benefit of this combination, demonstrating significant improvements in objective response rate (ORR), progression-free survival (PFS), and overall survival (OS) compared to platinum-based chemotherapy, with a median PFS of 12.5 versus 6.3 months and median OS of 31.5 versus 16.1 months, establishing EVP as the new standard first-line treatment. Despite these improvements in efficacy, EVP has a characteristic toxicity profile, with common adverse events including dermatologic adverse events, peripheral neuropathy, fatigue, and hyperglycemia [7].

Various studies have reported improved clinical outcomes among patients who develop certain treatment-related toxicities, suggesting a possible association between the occurrence of specific adverse events and treatment efficacy. In a large retrospective cohort study of 7008 patients treated with anti-PD-1 or anti-PD-L1 therapy, the development of dermatologic toxicities was associated with significantly improved OS compared with matched controls, with any cutaneous immune-related adverse event conferring a reduced risk of death (HR 0.778, 95% CI 0.726–0.834; *p* < 0.001), suggesting that dermatologic toxicity may reflect enhanced immune activation and treatment responsiveness [8]. Similarly, with EV, retrospective evidence suggests an association between treatment-emergent cutaneous toxicity and improved clinical outcomes [9]. Whether similar relationships exist with the combination of EVP, particularly for dermatologic toxicity, remains uncertain.

In this real-world study, we characterized the safety, tolerability, toxicity kinetics, and clinical outcomes of patients with la/mUC treated with EVP and explored whether treatment-related toxicities and delivered dose were associated with survival.

## 2. Methods

### 2.1. Study Design and Patients

We conducted a retrospective, multicenter cohort study of patients with la/mUC treated with first-line EVP in Alberta, Canada, between September 2024 and January 2026. Eligible patients were ≥18 years of age, had histologically confirmed urothelial carcinoma that was locally advanced/unresectable or metastatic, and received at least one cycle of EVP. Patients were required to have at least 3 months of clinical follow-up from treatment initiation; patients who died within three months remained eligible. Patients with a history of systemic treatment for locally advanced or metastatic disease were excluded from the study.

### 2.2. Data Collection

Patient demographics, disease characteristics, prior therapies, baseline laboratory parameters, treatment exposure, dose modifications, and clinical outcomes were extracted from electronic patient charts. Treatment-related peripheral neuropathy was defined as new-onset peripheral neuropathy after initiation of EVP or worsening of pre-existing peripheral neuropathy. The grading of treatment-related adverse events (AEs) was assessed according to CT CAE version 5.0. Treatment modifications were also documented, and categorized as dose reduction, treatment delay, or discontinuation with the underlying cause recorded when available.

### 2.3. Treatment

Each treatment cycle consisted of EV given intravenously on days 1 and 8, with a starting dose of either 1.25 mg/kg or 1 mg/kg, together with intravenous pembrolizumab 200 mg on day 1. EV dose-reduction levels used were 1.0, 0.75, and 0.5 mg/kg. Dose modifications were made at the treating physician’s discretion and according to institutional protocols.

## 3. Outcomes

The primary outcome assessed was treatment-related toxicity, including the incidence and severity of AEs, timing of AEs during treatment, and associated treatment modifications (frequency and pattern of dose reductions, delays, and discontinuations). Overall response rate (ORR), disease control rate (DCR), progression-free survival (PFS), and overall survival (OS) were secondary outcomes. Tumor response was assessed by the treating physicians according to RECIST v1.1, without central radiologic review. Radiological response assessments were performed after the first 3–4 treatment cycles and subsequently approximately every four cycles, according to routine clinical practice. Patients who experienced clinical deterioration before the first scheduled radiological assessment and therefore had no post-baseline imaging were considered non-evaluable for best radiological response. These patients remained included in the overall cohort for response analyses and were considered nonresponders when calculating ORR and as not achieving disease control when calculating DCR.

Time to AE onset was determined from date of first EVP dose to the date at which the treatment-related adverse event was first documented. Dermatologic toxicity and peripheral neuropathy were also evaluated independently to assess their relationship with clinical outcomes. The association between the initial EV dose (1 mg/kg vs. 1.25 mg/kg) and survival was also assessed.

## 4. Statistical Analysis

Baseline patient characteristics, treatment exposure, adverse events, and dose modifications were summarized using descriptive statistics. Categorical variables were presented as frequencies and percentages, whereas continuous variables were summarized using medians with ranges or interquartile ranges as appropriate. Among patients who developed treatment-emergent rash, a swimmer plot was used to descriptively illustrate the timing and grade of rash episodes in relation to EV and pembrolizumab exposure, rash-related treatment modifications, and treatment status at the end of follow-up. Comparisons of categorical outcomes were performed using either the chi-square test or Fisher’s exact test, as appropriate. The Kaplan–Meier method was used to evaluate PFS and OS. Cox proportional hazards regression was used to explore associations between treatment-related AEs, initial EV dose, and survival outcomes. Hazard ratios, odds ratios, and 95% confidence intervals were reported where applicable. A *p*-value < 0.05 was considered statistically significant, with all tests performed using a two-sided approach. Multivariable Cox proportional hazards analysis included prespecified baseline covariates, including age, histology, primary tumor site, lymph node status, visceral metastatic sites (lung, liver), bone mets and disease stage. The proportional hazards assumption was assessed using Schoenfeld residuals.

To account for potential immortal time bias, landmark analyses at 3 and 6 weeks after treatment initiation were performed to evaluate the association between early rash and OS. A landmark approach was selected because rash occurred early in the treatment course, allowing clinically relevant early time points to be prespecified while maintaining adequate numbers of patients and outcome events within this modest-sized cohort. Patients who were alive at the respective landmark were included, and rash status was classified according to whether rash had occurred on or before the landmark. Patients without rash or with rash developing after the landmark were classified as having no early rash. Overall survival was measured from the landmark onward using Cox proportional hazards regression.

Analyses were performed using SPSS version 29.0 (IBM Corp, Armonk, NY, USA).

## 5. Results

### 5.1. Patient Characteristics

**Figure 1:**
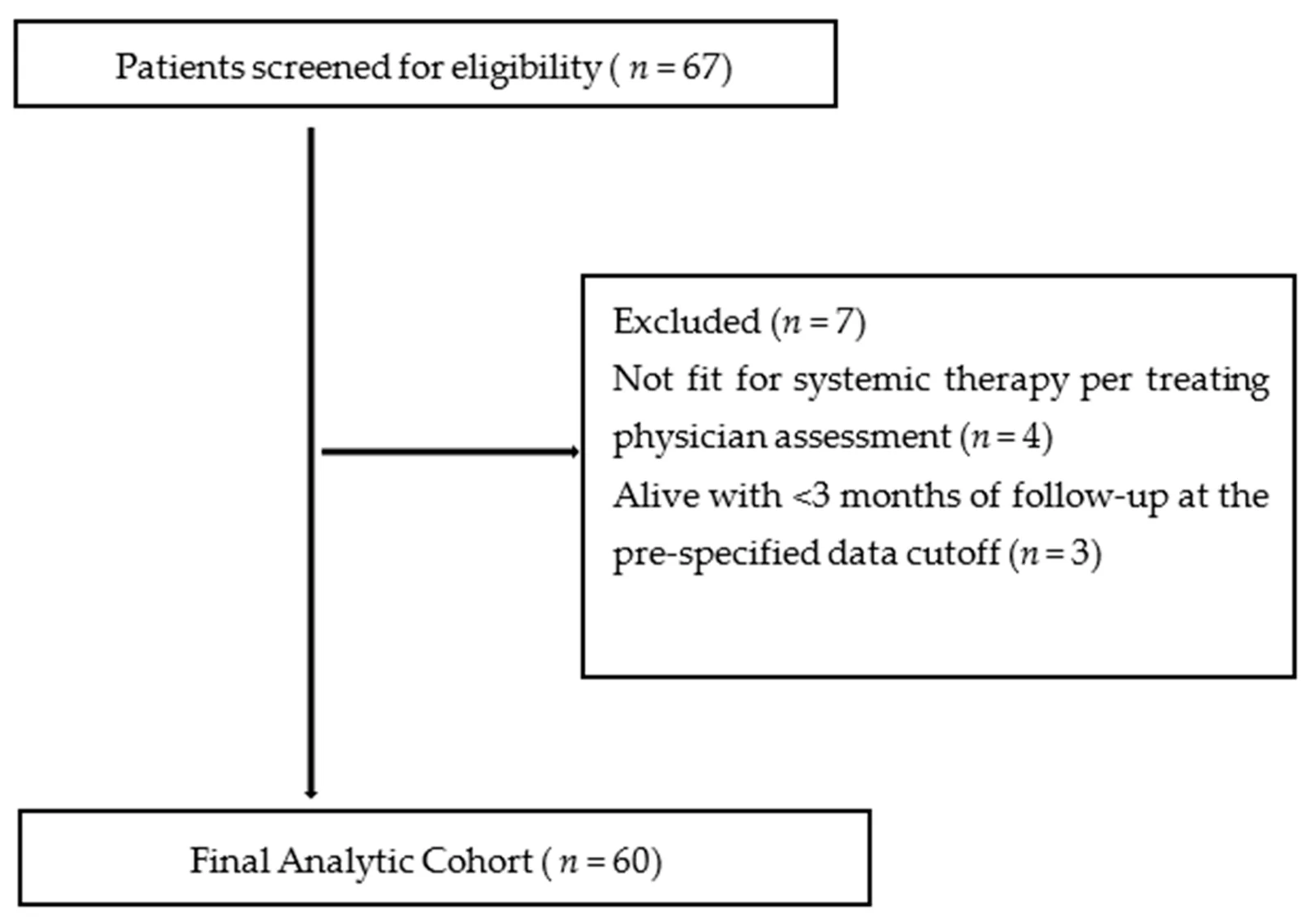
Flow diagram of patient selection and study cohort.

A total of 67 patients were screened for eligibility, of whom 60 were included in the final analysis (Figure 1).

Sixty patients were included. Median age was 69 years, and 82% were male. The primary tumor site was bladder in 72% and upper urinary tract in 23%. Baseline peripheral neuropathy was present in six patients (10%), and all were grade 1. Most patients had pure urothelial histology (82%), while 18% had mixed histology. At treatment initiation, 45% had visceral metastases, 25% had non-regional lymph node-only disease, and 20% had locally advanced/unresectable disease. Prior neoadjuvant cisplatin–gemcitabine had been administered in six patients (10%), and 14 patients (23%) had undergone prior cystectomy or nephroureterectomy (Table 1).

### 5.2. Treatment Exposure

**Figure 2:**
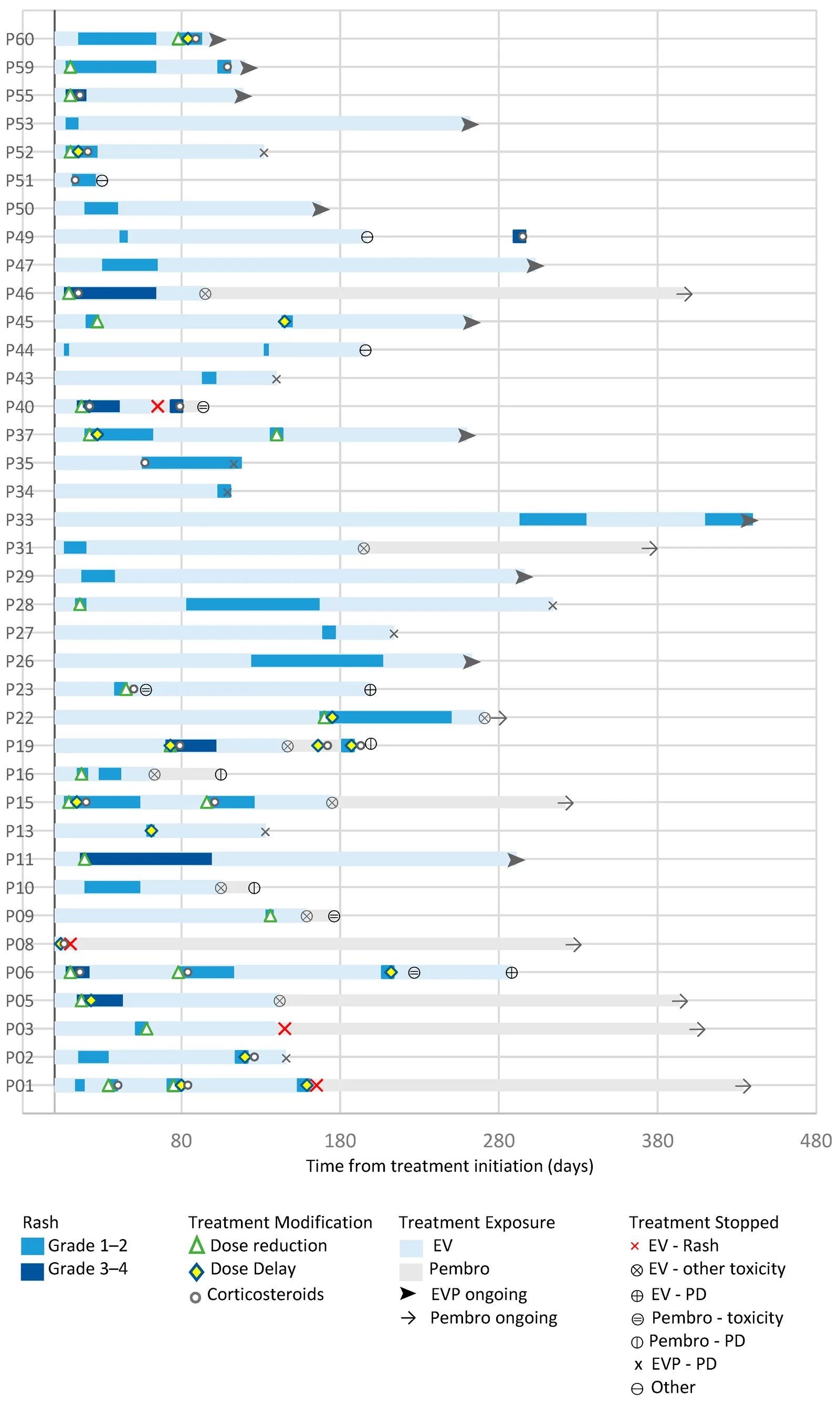
Swimmer plot illustrating treatment exposure and treatment-emergent rash among patients who developed rash (*n* = 38). Bars represent the duration of EV and pembrolizumab exposure, with rash episodes shown according to timing and grade. Symbols indicate rash-related treatment modifications, including dose reductions, treatment delays, and corticosteroid use. Arrows indicate ongoing treatment at the end of follow-up.

The majority of patients (77%) initiated EV at 1.25 mg/kg. Baseline characteristics according to initial EV dose are presented in Supplementary Table S1. The median number of EV and pembrolizumab treatment cycles administered was seven (IQR, 5–9.25) and eight (IQR, 5–13), respectively. Dose reductions occurred in 62% of patients and treatment delays in 43%. Treatment discontinuation occurred in 38% (EV alone 27%, pembrolizumab alone 8%, both 3%) (Table 2). Day 8 EV dose omissions could not be reliably differentiated from treatment delays because of the retrospective nature of data collection. Among patients who developed rash, the median time to first rash was 17 days (IQR 11.5–57.3), with 60.5% occurring within 3 weeks and 81.6% being grade 1–2 at first occurrence. Rash characteristics, management, and treatment exposure are summarized in Supplementary Table S7 and illustrated in swimmer plots (Figure 2).

The median number of EV cycles before discontinuation was five for rash (IQR, 2.5–7.25), five for fatigue (IQR, 4.75–6.75), and six for peripheral neuropathy (IQR, 6–8).

### 5.3. Treatment-Related Adverse Events

**Figure 3:**
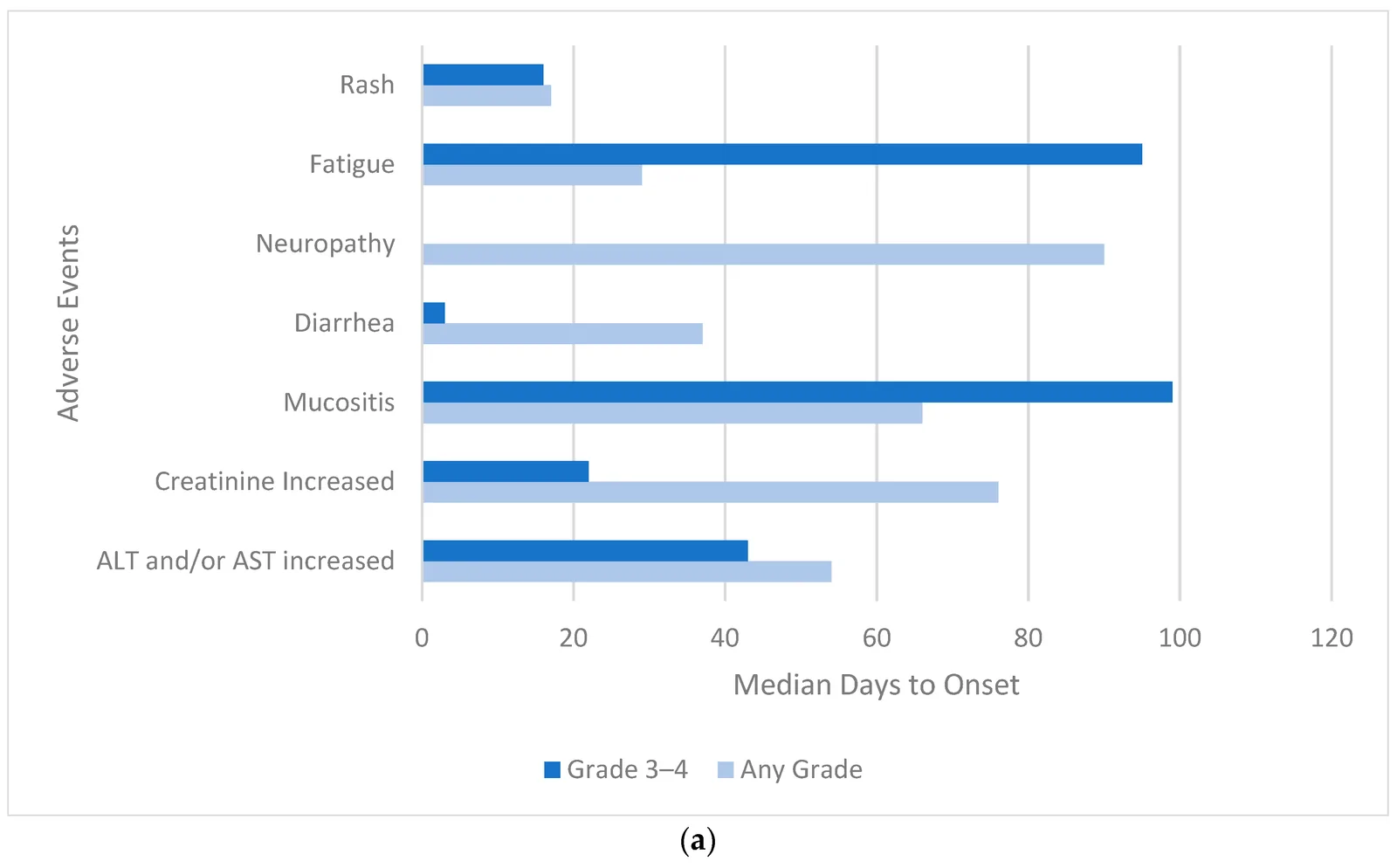

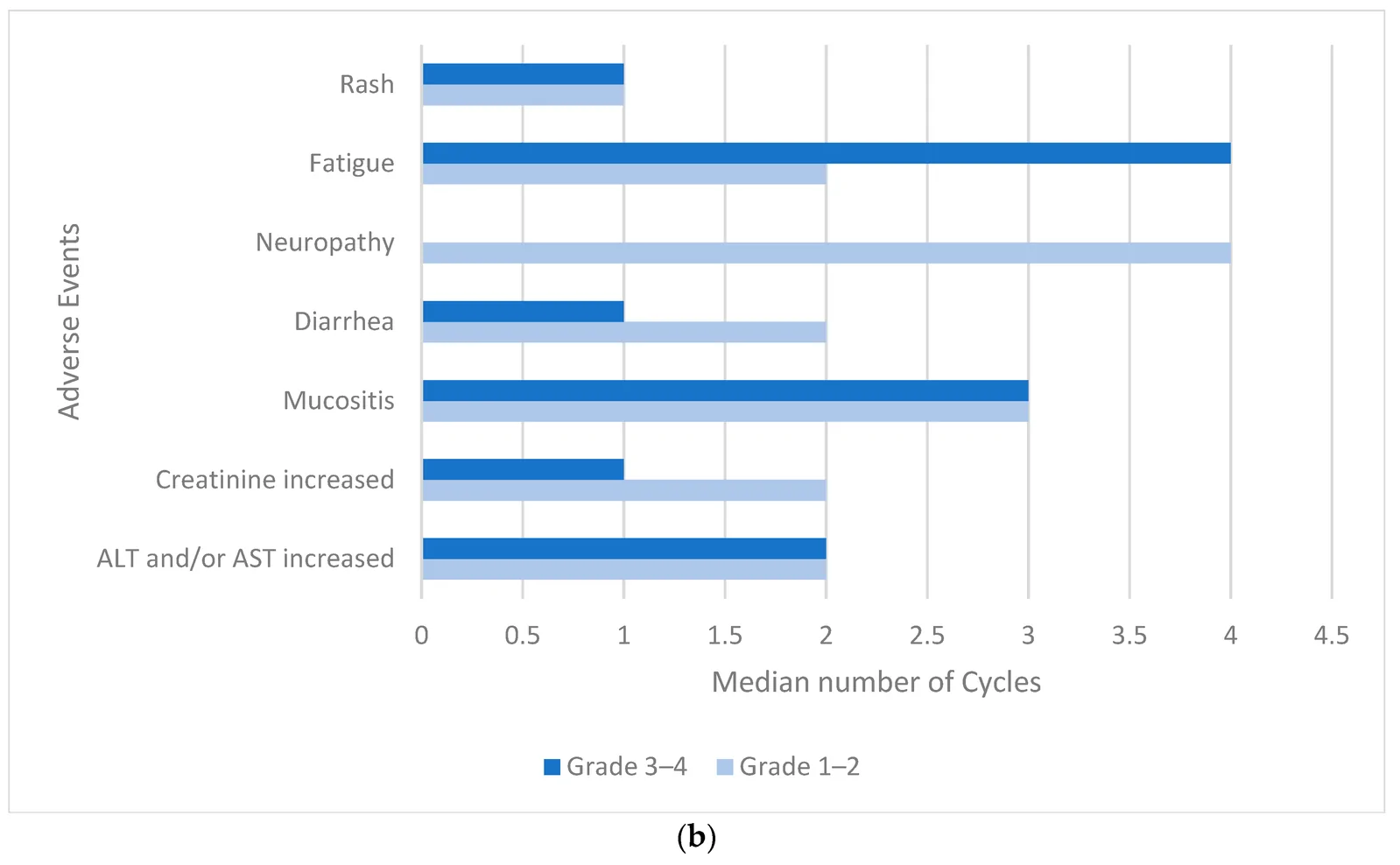
(**a**) Median time to onset in days for adverse events of any grade and Grade 3–4. (**b**) Median number of treatment cycles to onset of treatment-related adverse events of any grade and Grade 3–4.

The most common treatment-related AEs of any grade were rash (*n* = 38, 63%), fatigue (*n* = 34, 57%), and peripheral neuropathy (*n* = 28, 47%). Grade 3–4 AEs included rash (15%), fatigue (8%), and mucositis (3%). The timing of toxicity varied by event type. Rash occurred earliest, with a median onset of 17 days, followed by fatigue at 29 days and neuropathy at 90 days. Thromboembolic events occurred in 10% of patients (Table 3, Figure 3a,b). Patients who developed rash or peripheral neuropathy had greater treatment exposure than those who did not (Supplementary Table S6).

Overall, nine patients (15%) required hospitalization for treatment-related toxicities, including three for rash, two for presumed immune-mediated colitis, and one each for presumed immune-mediated mucositis/esophagitis, pneumonitis, myocarditis/nephritis, and myositis. No treatment-related deaths were observed. Baseline characteristics according to the development of treatment-emergent rash and peripheral neuropathy are presented in Supplementary Table S2.

### 5.4. Efficacy

After a median follow-up of 10.3 months, 11 of 60 patients (18%) achieved a complete response (CR), 26 (43%) had a partial response (PR), and 15 (25%) had stable disease (SD). Four patients (7%) had progressive disease (PD) at the first radiological assessment, while four (7%) experienced clinical deterioration before the first scheduled radiological assessment and therefore had no post-baseline imaging. The objective response rate (ORR) was 61.7%, and the disease control rate (DCR) was 86.7%. At the time of analysis, median PFS was 10.8 months (95% CI, 8.1 months—not reached), while median OS had not yet been reached. At 1 year, the estimated PFS rate was 41.1% (95% CI, 27.0–62.6), and the estimated OS rate was 63.3% (95% CI, 47.7–84.2).

### 5.5. Association Between Treatment-Related AEs and Outcomes

**Figure 4:**
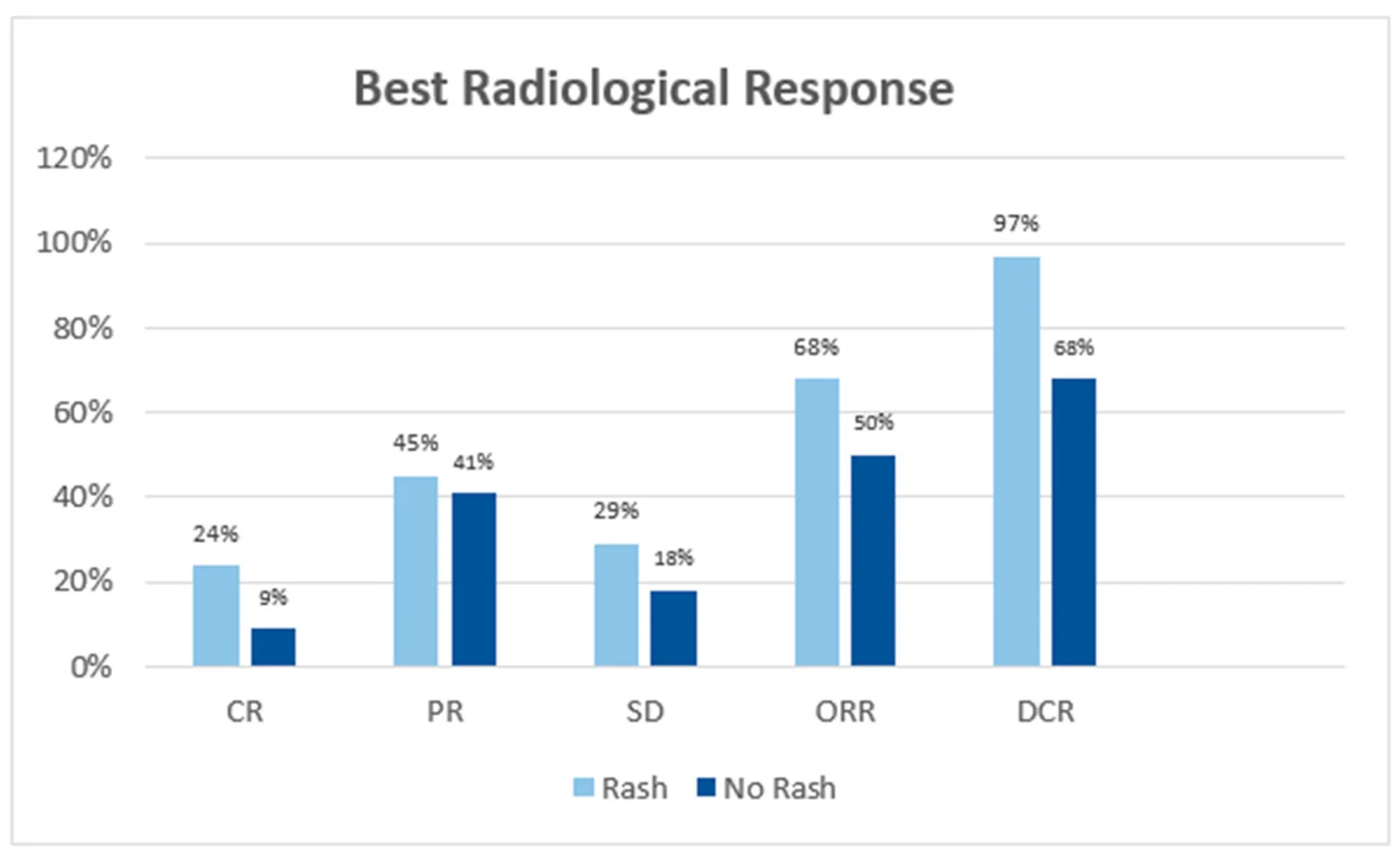
Rash and its association with response.

The ORR was 68.4% among patients who developed rash compared with 50% among those who did not, although the difference was not statistically significant (OR 2.17, 95% CI 0.74–6.38; *p* = 0.157), whereas DCR was significantly higher among patients who developed rash (97.4% vs. 68.2%; OR 17.27, 95% CI 1.95–152.7; *p* = 0.003) (Figure 4).

**Figure 5:**
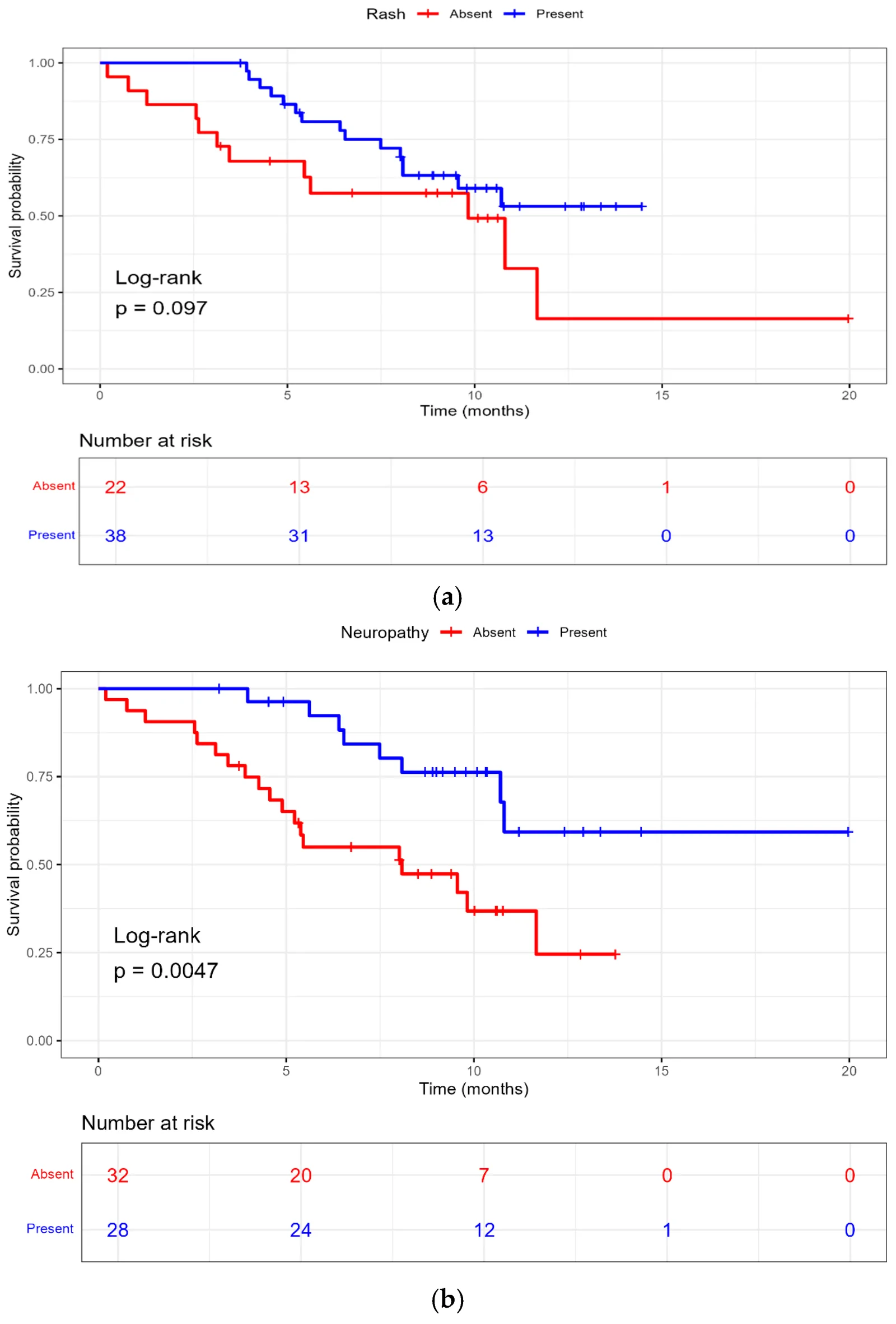

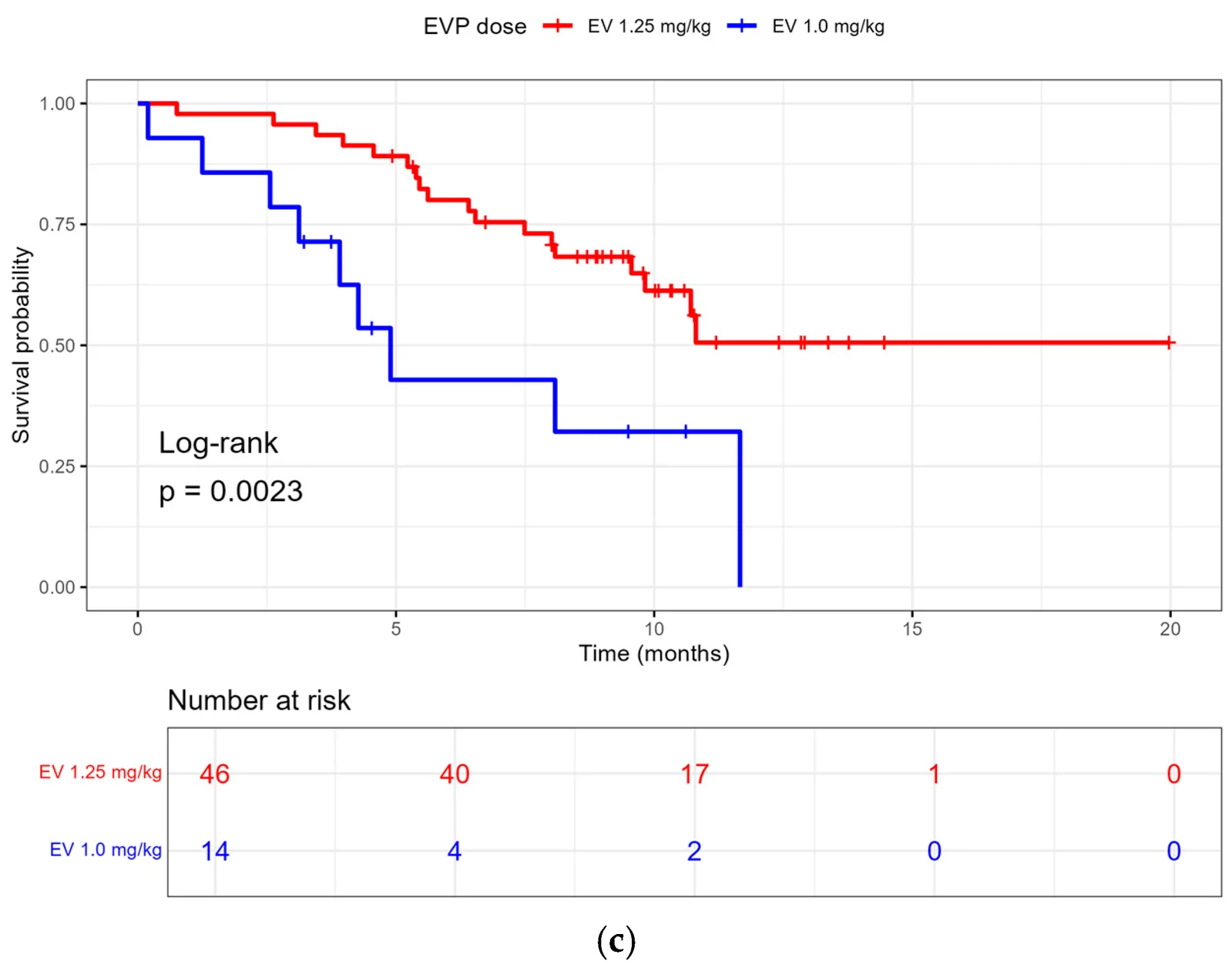
Kaplan–Meier analyses of progression-free survival according to treatment-related adverse events and initial EV dose. (**a**) Rash and progression-free survival. (**b**) Neuropathy and progression-free survival. (**c**) EVP dose and progression-free survival.

**Figure 6:**
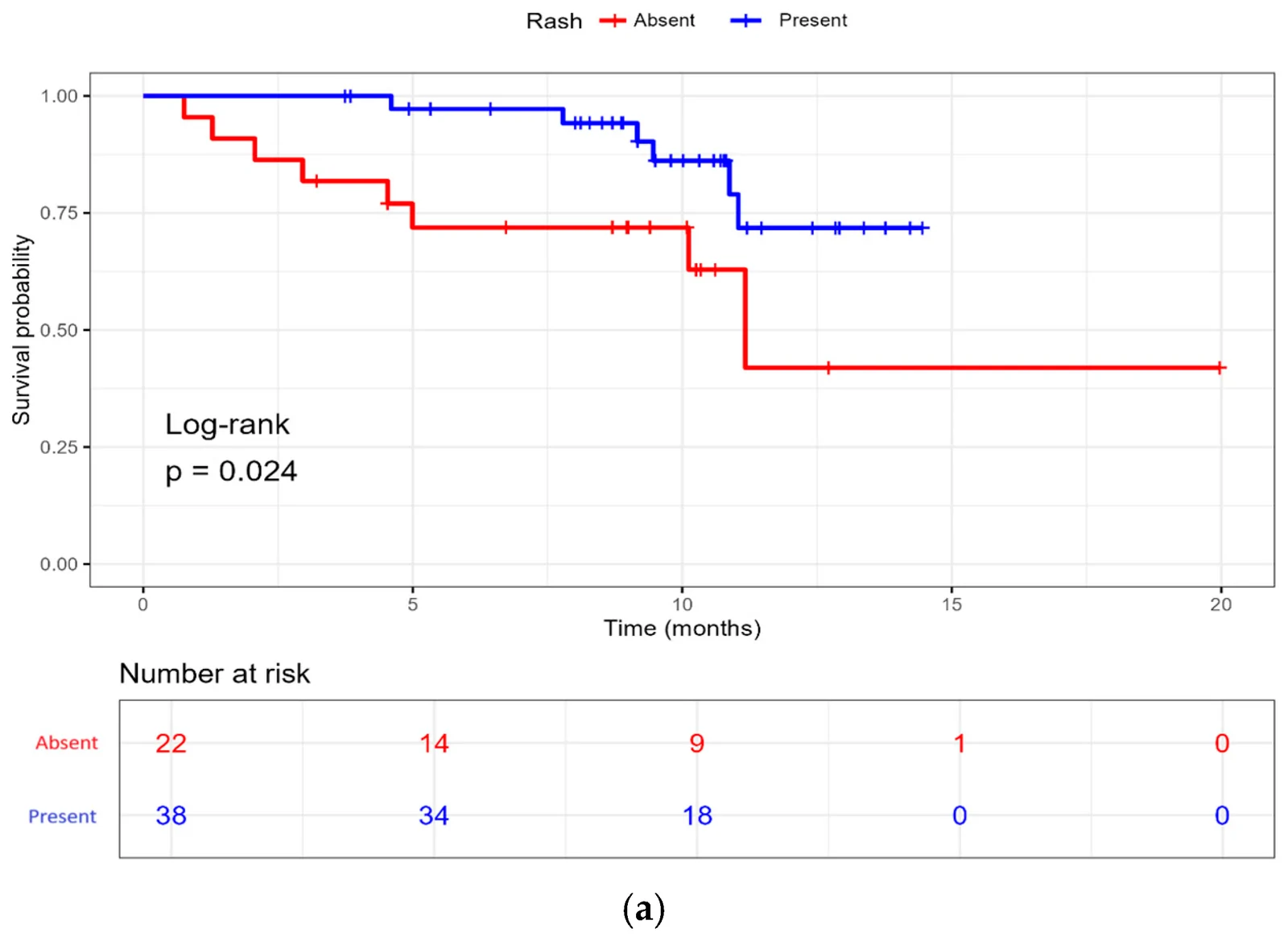

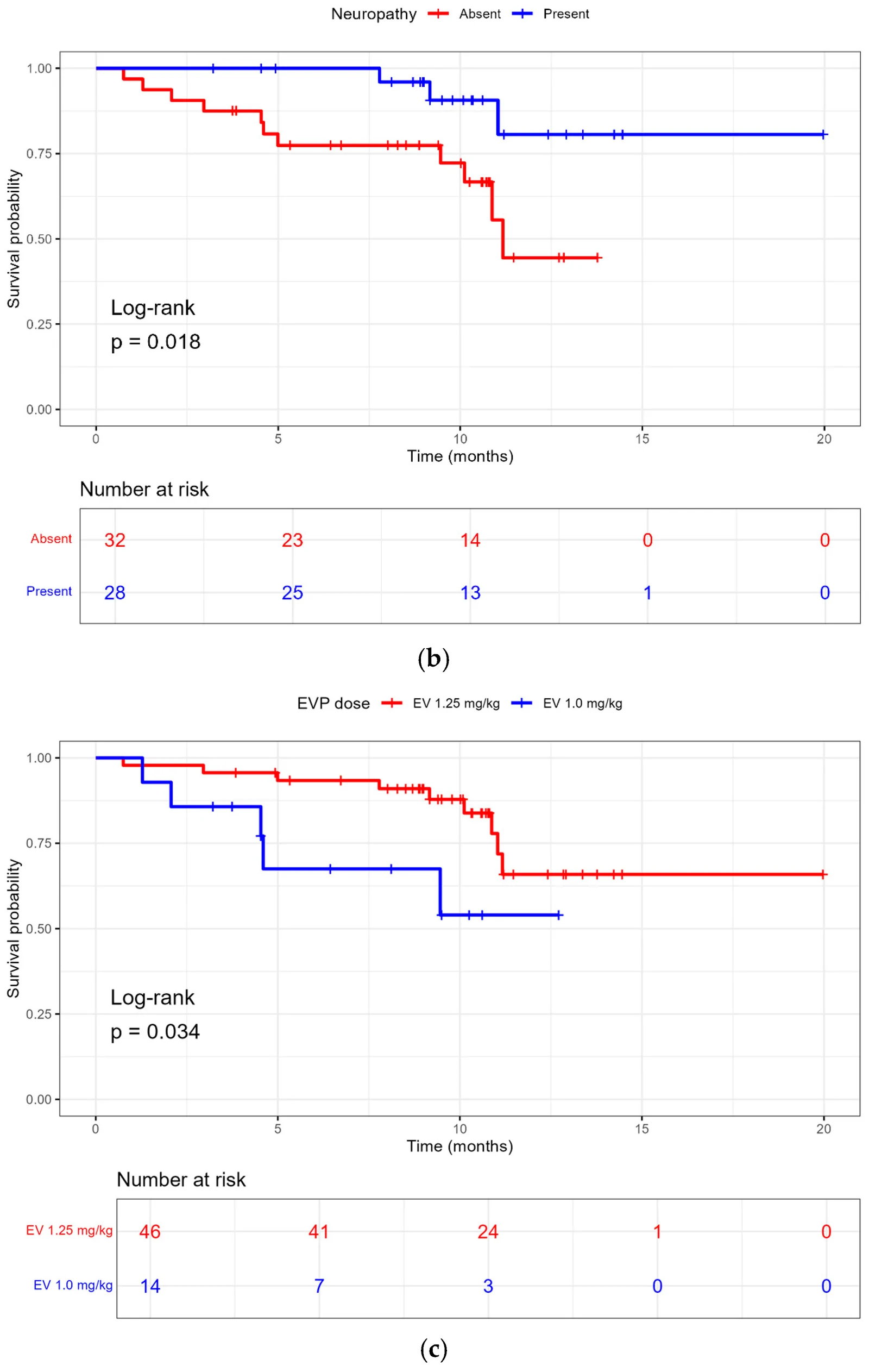
Kaplan–Meier analyses of overall survival according to treatment-related adverse events and initial EV dose. (**a**) Rash and overall survival. (**b**) Neuropathy and overall survival. (**c**) EVP dose and overall survival.

Among patients who developed rash, median PFS was not reached (NR), compared with 9.8 months among those without rash. This difference did not reach statistical significance (HR 0.53, 95% CI 0.25–1.14; *p* = 0.10) (Figure 5a). In contrast, median overall survival (OS) was NR versus 11.2 months, with treatment-related rash associated with a significantly lower risk of death (HR 0.31, 95% CI 0.11–0.91; *p* = 0.032) (Figure 6a).

Landmark analyses were performed at 3 and 6 weeks to evaluate the association between early rash and OS while accounting for immortal time bias. At the 3-week landmark, early rash was associated with a lower hazard of death, although the association did not reach statistical significance (HR 0.25, 95% CI 0.06–1.13; *p* = 0.07). A similar association was observed at the 6-week landmark (HR 0.26, 95% CI 0.06–1.18; *p* = 0.08). The number of post-landmark deaths was limited (14 after the 3-week landmark and 12 after the 6-week landmark), resulting in wide confidence intervals (Supplementary Table S5).

Peripheral neuropathy was also associated with improved outcomes. Median PFS was significantly longer among patients who developed treatment-related neuropathy than among those who did not (NR vs. 8.1 months; HR 0.32, 95% CI 0.14–0.73; *p* = 0.007) (Figure 5b). Median OS was also significantly longer among patients who developed neuropathy (NR vs. 11.2 months; HR 0.24, 95% CI 0.07–0.87; *p* = 0.029) (Figure 6b).

In multivariable analysis, only neuropathy was independently associated with improved PFS (HR 0.18, 95% CI 0.06–0.52 *p* = 0.002), while both neuropathy and rash were independently associated with improved OS (neuropathy: HR 0.11, 95% CI 0.02–0.54; *p* = 0.007; rash: HR 0.26, 95% CI 0.07–0.93; *p* = 0.039), after adjustment for baseline clinical and disease characteristics (Supplementary Tables S3 and S4).

### 5.6. Initial EV Dose and Outcomes

EV initiation at 1.25 mg/kg was associated with improved PFS compared with 1.0 mg/kg on univariable analysis (HR 0.30, 95% CI 0.13–0.68; *p* = 0.004) (Figure 5c), and the association remained significant on multivariable analysis (HR 0.26, 95% CI 0.08–0.86; *p* = 0.027). Similarly, EV initiation at 1.25 mg/kg was associated with improved OS on univariable (HR 0.32, 95% CI 0.10–0.97; *p* = 0.044) (Figure 6c) and multivariable analyses (HR 0.19, 95% CI 0.04–0.94; *p* = 0.042) (Supplementary Tables S3 and S4).

There was no evidence of violation of the proportional hazards assumption for the individual covariates or for the overall Cox proportional hazards model (global test, *p* > 0.05).

## 6. Discussion

Our multicenter real-world analysis of first-line EVP in patients with locally advanced or metastatic urothelial carcinoma demonstrated clinical outcomes comparable to those observed in prospective studies. The observed ORR of 61.7% and DCR of 86.7% support the effectiveness of this regimen in routine practice. These findings complement the results of the EV-302/KEYNOTE-A39 trial, and its longer-term follow-up, which continued to show substantial clinical benefit with first-line EVP [7,10].

The pattern of treatment-related toxicities observed in our cohort was similar to that reported in earlier real-world studies of EVP. In a previously reported real-world analysis of 183 patients, treatment-related adverse events occurred in 94% of patients, with grade ≥ 3 events in 28%. Dermatologic toxicity (55%), peripheral neuropathy (40%) and fatigue (40%) were among the most frequently reported adverse events, often leading to dose reductions (46%) and treatment discontinuation (32%) [11]. In this analysis, a similar AE profile was noted. Rash, fatigue, and peripheral neuropathy were the most frequently observed adverse events, with dermatologic events accounting for most grade 3–4 toxicities. The onset of toxicity varied according to the type of adverse event observed. Rash generally developed early in the treatment course, with a median time to onset of 17 days, whereas peripheral neuropathy tended to appear later, reflecting a cumulative toxicity pattern. These differences are clinically relevant, as close attention to dermatologic symptoms and early patient education may allow dermatologic toxicity to be addressed promptly and may help prevent further worsening, whereas assessment for neuropathy should continue throughout the course of treatment.

In this analysis, dose modifications were frequent, with dose reductions occurring in 62% of patients, treatment delays in 43%, and treatment discontinuation in 38%. These findings highlight the need for active toxicity surveillance and individualized treatment adjustment in routine practice. Although dose modifications are often required, they should not be interpreted as treatment failure. Rather, they reflect the practical management required to maintain patients on an effective regimen while potentially minimizing clinically significant toxicity.

One of the exploratory findings from our study was that patients who developed treatment-related rash had better clinical outcomes. Patients who developed rash had significantly higher DCR and improved OS compared with those who did not. Although ORR was numerically higher among patients with rash, this difference did not reach statistical significance. Similar findings have been reported in a retrospective study of patients receiving EV, where cutaneous toxicity was associated with better DCR and OS [12]. Our findings suggest that dermatologic toxicity may serve as an exploratory on-treatment correlate of drug exposure, pharmacodynamic effect, or underlying biological sensitivity to EV-based therapy.

The mechanism linking rash with improved outcomes remains uncertain. EV-associated dermatologic toxicity may reflect on-target effects related to nectin-4 expression in normal epidermal structures, systemic exposure to the antibody–drug conjugate payload, or immune-mediated mechanisms potentiated by pembrolizumab [13]. Emerging translational data suggest that immune processes, including autoantibody responses, may contribute to the development of EV-related cutaneous toxicity [14]. Together, these observations support further investigation of rash as a potential pharmacodynamic biomarker, while recognizing that its presence should not be considered a validated surrogate for response.

Patients who developed peripheral neuropathy also appeared to have better PFS and OS in our cohort. This finding should be interpreted cautiously, as neuropathy often develops later during therapy and may therefore be influenced by treatment duration and survivorship bias. Patients who remain on treatment longer have greater opportunity both to benefit from therapy and to develop cumulative toxicity. Nonetheless, the association between neuropathy and improved outcomes is hypothesis-generating and reinforces the importance of evaluating toxicity kinetics alongside efficacy endpoints in future studies.

Importantly, to account for the time-dependent nature of treatment-emergent toxicities and mitigate immortal time bias, landmark analyses were performed at 3 and 6 weeks for rash. Early rash demonstrated a consistent association with improved overall survival across both landmark time points (3-week landmark: HR 0.25, 95% CI 0.06–1.13; *p* = 0.07; 6-week landmark: HR 0.26, 95% CI 0.06–1.18; *p* = 0.08), with a stable direction and magnitude of effect despite limited statistical power. The limited number of events at each time point for landmark analysis resulted in imprecise estimates with wide confidence intervals and reduced statistical precision. Given the small sample size and limited number of outcome events, these observations should be considered exploratory and primarily hypothesis-generating. Landmark analyses were restricted to rash because of its early onset (median 17 days), whereas the substantially later onset of neuropathy (median 90 days) and the limited number of events precluded a meaningful landmark analysis.

Initial EV dosing appeared to be associated with clinical outcomes. Patients who initiated therapy at 1.25 mg/kg had superior progression-free survival compared with those who began at 1.0 mg/kg. On univariable Cox analysis, initiation at 1.25 mg/kg was associated with a lower risk of progression and death, and these associations remained significant on multivariable analysis. However, given the small number of patients initiated at 1.0 mg/kg and the potential for confounding by indication and other baseline clinical factors, these findings should be interpreted cautiously. Patients selected for lower starting doses may have had greater frailty, comorbidity burden, poorer performance status, renal dysfunction, or other adverse prognostic features independent of treatment exposure. Nevertheless, these findings suggest that upfront dose reduction should be approached thoughtfully, and prospective studies are needed to clarify whether early dose intensity independently influences outcomes with EVP.

Our study has several limitations. Given its retrospective nature, there is a possibility of selection bias, missing data, and differences in how adverse events were documented in clinical practice. Treatment decisions, dose modifications, timing of imaging, and follow-up were based on routine clinical care and were not standardized. The relatively small sample size limited the subgroup and exploratory analyses. Follow-up was also short and may not have captured late toxicities, particularly peripheral neuropathy, or longer-term survival outcomes. Although multivariable analyses were adjusted for relevant baseline factors, the small number of outcome events may have resulted in model overfitting and less precise estimates. A time-dependent Cox analysis was not performed because of the modest sample size and limited number of OS events. Although the landmark analyses were used to account for potential immortal time bias, they do not incorporate the full timing of AE onset in the same manner as a time-dependent model. Therefore, the observed associations should be considered exploratory and require confirmation in larger cohorts.

Nevertheless, these findings remain clinically important, as they provide early real-world insight into the tolerability, dosing patterns, and potential efficacy implications of EVP outside of a clinical trial setting. Given the increasing adoption of this regimen in routine practice, real-world data are essential to inform patient selection, dosing strategies, and toxicity management. An exploratory signal of an association between treatment-emergent rash and improved outcomes was observed; however, this finding remains vulnerable to time-dependent and residual confounding and requires validation in larger cohorts. Ongoing follow-up of this cohort will allow for more mature survival analyses, improved characterization of cumulative and late toxicities, and further evaluation of the relationship between dose intensity and clinical outcomes.

## 7. Conclusions

EVP demonstrated substantial real-world efficacy and acceptable tolerability in patients with advanced urothelial carcinoma. Early dermatologic toxicity was associated with improved clinical outcomes, suggesting a potential role as an on-treatment marker of treatment activity. Prospective studies are warranted to further validate toxicity–efficacy relationships, clarify the impact of dose intensity, and optimize patient selection and management.

## Figures and Tables

**Table 1:** Baseline demographics.

Characteristics	*n* = 60 (%)
Gender	
Male	49 (81.7%)
Female	11 (18.3%)
Median age (range)—years	69 (46–86)
Site of primary	
Bladder	43 (71.7%)
Upper genitourinary tract	14 (23.3%)
Both	3 (5%)
Baseline neuropathy ^a^	6 (10%)
ECOG	
0–1	45 (75%)
2–4	15 (25%)
Histology	
Pure urothelial	49 (81.7%)
^b^ Mixed histology	11 (18.3%)
Squamous differentiation	4 (6.7%)
Multiple variant components	4 (6.7%)
Neuroendocrine component	1 (1.7%)
Micropapillary component	1 (1.7%)
Plasmacytoid component	1 (1.7%)
TNM stage at diagnosis	
T status	
Ta/Tis	12 (20%)
T1/T2	29 (48.3%)
T3/T4	12 (20%)
Tx	7 (11.7%)
Nodal status	
N0/N1	35 (58.3%)
N2/N3	22 (36.7%)
Nx	3 (5%)
Disease category at diagnosis	
Non-muscle invasive	22 (36.7%)
Muscle invasive	25 (41.7%)
Metastatic	13 (21.7%)
Disease category at treatment initiation	
Locally advanced	12 (20%)
Metastatic disease	48 (80%)
One site	29 (48.3%)
Two sites	15 (25%)
More than two sites	4 (6.7%)
Sites of metastases ^c^	
Visceral	27 (45%)
Bone	12 (20%)
Lung	16 (26.7%)
Liver	12 (20%)
Other	5 (8.3%)
Lymph node only (non-regional)	15 (25%)
Received prior neoadjuvant therapy for MIBC	
Cisplatin + gemcitabine	6 (10%)
Prior curative surgery	
Radical cystectomy	7 (11.7%)
Radical nephroureterectomy	7 (11.7%)
None	46 (76.7%)
Initial dose of EV + pembrolizumab	
EV 1.25 mg/kg + pembrolizumab 200 mg	46 (76.7%)
EV 1 mg/kg + pembrolizumab 200 mg	14 (23.3%)
FGFR status	
Positive	8 (13.3%)
Negative	23 (38.3%)
Unknown	29 (48.3%)

Abbreviations: MIBC, muscle-invasive bladder cancer; EV, enfortumab vedotin; FGFR, fibroblast growth factor receptor; ECOG, Eastern Cooperative Oncology Group. ^a^ Baseline peripheral neuropathy was present in 6/60 patients (10%), all grade 1. The underlying cause was undocumented in four patients, while one had degenerative disc disease and one had systemic sclerosis. ^b^ All mixed-histology tumors contained a urothelial carcinoma component. Four tumors contained multiple variant components: squamous/glandular/giant cell (*n* = 1), squamous/plasmacytoid/giant cell (*n* = 1), glandular/squamoid (*n* = 1), and extensive squamous/sarcomatoid (*n* = 1). One tumor had neuroendocrine features with focal pseudoglandular morphology. ^c^ Visceral metastases were defined as visceral involvement without bone metastases. Bone, lung, liver, and other metastatic sites were not mutually exclusive.

**Table 2:** Treatment modifications according to treatment related toxicity.

Toxicity	EV Dose Reduction ^a^	Temporary Hold/Delay	Permanent Discontinuation			Hospitalization
			EV	Pembro	Both	
Overall	37 (61.7%)	26 (43.3%)	16 (26.7%)	5 (8.3%)	2 (3.3%)	9 (15%)
Rash ^b^	20 (33.3%)	14 (23.3%)	4 (6.7%)	1 (1.7%)		3 (5%)
Neuropathy ^b^	12 (20%)	3 (5%)	6 (10%)	-	1 (1.7%)	0
Fatigue ^b^	14 (23.3%)	5 (8.3%)	4 (6.7%)	-	-	0
Other ^c^	5 (8.3%)	10 (16.7%)	2 (3.3%)	5 (8.3%)	1 (1.7%)	6 (10%)

^a^ Dose reduction refers exclusively to EV. Reduced EV dose levels used were 1.0, 0.75, and 0.5 mg/kg; patients could undergo sequential dose reductions during treatment. ^b^ Rash, neuropathy, and fatigue were not mutually exclusive reasons for treatment modification; individual patients could have more than one toxicity contributing to a treatment modification. Therefore, toxicity-specific counts may overlap and do not sum to the overall number of patients with treatment modifications. ^c^ Other toxicities resulting in EV dose reduction included nausea/decreased appetite (*n* = 3), diarrhea (*n* = 1), and elevated AST/ALT (*n* = 1). Other toxicities resulting in treatment hold/delay included diarrhea (*n* = 2), elevated AST/ALT (*n* = 2), mucositis (*n* = 1), myocarditis (*n* = 1), elevated creatine kinase (*n* = 1), pruritus (*n* = 1), adrenal insufficiency (*n* = 1), and hyponatremia (*n* = 1). Other toxicities resulting in permanent treatment discontinuation included colitis (*n* = 2), mucositis (*n* = 1), myocarditis (*n* = 1), and pneumonitis (*n* = 1) for pembrolizumab; mucositis (*n* = 1) and decreased appetite/nausea (*n* = 1) for EV; and hypotensive shock (*n* = 1) for both agents.

**Table 3:** Treatment-related adverse events.

	*n* = 60 (%)
Any grade	58 (96.7%)
Grade III–IV	18 (30%)
Rash	38 (63%)
G I–II	29 (48.3%)
G III–IV	9 (15%)
Neuropathy	28 (46.7%)
G I–II	28 (46.7%)
G III–IV	
Fatigue	34 (56.7%)
G I–II	29 (48.3%)
G III–IV	5 (8.3%)
AST and/or ALT increased	14 (23.3%)
G I–II	13 (21.7%)
G III–IV	1 (1.7%)
Creatinine increased	13 (21.7%)
Grade I–II	12 (20%)
Grade III–IV	1 (1.7%)
Mucositis	4 (6.7%)
G I–II	2 (3.3%)
G III–IV	2 (3.3%)
Hyperglycemia	10 (16.7%)
G I–II	10 (16.7%)
G III–IV	
Diarrhea (immunotherapy-related ^a^)	5 (8.3%)
G I–II	4 (6.7%)
G III–IV	1 (1.7%)
Pneumonitis (immunotherapy-related)	1 (1.7%)
G I–II	1 (1.7%)
Myocarditis (immunotherapy-related)	1 (1.7%)
Elevated CK (Immune)	6 (10%)
Grade I–II	4 (6.7%)
Grade III–IV	2 (3.3%)
Thromboembolic events	6 (10%)
DVT	1 (1.7%)
PE	5 (8.3%)

^a^ Drug-specific attribution was based on the treating physician’s documented assessment; where attribution between EV and pembrolizumab could not be reliably determined, it was not assigned to an individual agent.

## Data Availability

The data presented in this study are available on request from the corresponding author. Data are not publicly available due to privacy restrictions.

## Supplementary Materials

The following supporting information can be downloaded at: https://www.mdpi.com/article/10.3390/curroncol33090542/s1, Table S1: Baseline characteristics according to starting EV dose. Table S2: Baseline characteristics according to treatment-emergent rash and peripheral neuropathy. Table S3: Univariate and multivariable Cox proportional hazards analyses for progression-free survival. Table S4: Univariate and multivariable Cox proportional hazards analyses for overall survival. Table S5: Landmark analyses of early rash and overall survival. Table S6: Treatment exposure according to treatment-emergent rash and peripheral neuropathy. Table S7: Characteristics, management, and treatment exposure among patients who developed treatment-emergent rash.
