# Supplementary material for: Toxicity, Dose Intensity, and Clinical Outcomes with First-Line Enfortumab Vedotin Plus Pembrolizumab in Advanced Urothelial Carcinoma: A Multicenter Real-World Study
Source: Curr Oncol. 2026 Sep 9;33(9):542. doi: 10.3390/curroncol33090542 (PMC13605319; doi:10.3390/curroncol33090542)
Supplement: Supplementary file 1 [file curroncol-33-00542-s001.zip › curroncol-4503146-supplementary.pdf]

Supplementary Materials

Supplementary Table S1. Baseline characteristics according to starting EV dose

Characteristics	EV 1.25mg/Kg (n=46)	EV 1mg/kg[a] (n = 14)
Age median(IQR)	68 (63.3–72.0)	73 (66.0–74.0)
Male sex, n (%)	37 (80.4%)	12 (85.7%)
Hemoglobin, g/L, median (IQR)	126 (112.8–141.5)	123.5 (107–131)
Creatinine clearance, mL/min, median (IQR)	65 (49.5–83.8)	52 (41–81)
Hypertension n (%)	19 (41.3%)	5 (35.7%)
Diabetes mellitus, n (%)	10 (21.7%)	3 (21.4%)
Ischemic Heart Disease, n (%)	10 (21.7%)	4 (28.6%)
Prior neoadjuvant chemotherapy (platinum/gemcitabine)	3 (6.5%)	3 (21.4%)
Metastatic Disease at EVP initiation	35 (76.1%)	13 (92.8%)
Baseline peripheral neuropathy	5 (10.9%)	1 (7.1%)
ECOG		
0-1	37 (80.4%)	8 (57.1%)
2-4	9 (19.6%)	6 (42.9%)

[a]A specific rationale for reduced-dose initiation was documented in 6/14 patients (42.9%), including baseline neuropathy (n=1), advanced age (n=1), connective tissue disease/interstitial lung disease (n=1), recent cord compression/spinal hardware infection (n=1), ulcerative colitis (n=1), and a pelvic tumor–sigmoid fistula with concern for infection (n=1). In the remaining 8 patients (57.1%), no specific clinical rationale was documented, although six were noted to have been started at a reduced dose to assess treatment tolerance based on physician discretion.

Supplementary Table S2. Baseline characteristics according to treatment-emergent rash and peripheral neuropathy

Characteristics	Rash	No Rash	Neuropathy	No Neuropathy
Age median(IQR)	70.5 (66.0–76.5)	66.0 (63.0–72.0)	68.0 (59.0–74.3)	71.0 (66.0–74.0)
Male sex, n (%)	32 (84.2%)	17 (77.3%)	21 (75.0%)	28 (87.5%)
Hemoglobin, g/L, median (IQR)	128.5 (115.3–139.5)	122.0 (106.0–135.5)	132.0 (118.5–143.8)	121.5 (102.5–132.5)
Creatinine clearance, mL/min, median (IQR)	56.5 (47.5–83.0)	63.5 (48.3–82.3)	66.5 (53.5–84.5)	51.5 (39.0–78.8)
Hypertension n (%)	19 (50%)	5 (22.7%)	13 (46.4%)	11 (34.4%)
Diabetes mellitus, n (%)	8 (21.1%)	5 (22.7%)	2 (7.1%)	11 (34.4%)
Ischemic Heart Disease, n (%)	10 (26.3%)	4 (18.2%)	6 (21.4%)	8 (25.0%)
Prior neoadjuvant chemotherapy (platinum/gemcitabine)	4 (10.5%)	2 (9.1%)	5 (17.9%)	1 (3.1%)
Metastatic Disease at EVP initiation	30 (78.9%)	18 (81.8%)	24 (85.7%)	24 (75.0%)
ECOG				
0-1	29 (76.3%)	16 (72.7%)	22 (78.6%)	23 (71.9%)
2-4	9 (23.7%)	6 (27.3%)	6 (21.4%)	9 (28.1%)

Supplementary Table S3. Univariate and multivariable Cox proportional hazards analyses for progression-free survival.

A. Model including treatment-emergent rash

	Univariate			Multivariable		
**Characteristic**	**HR**	**95% CI**	**p-value**	**HR**	**95% CI**	**p-value**
**Rash**						
Absent	—	—		—	—	
Present	0.53	0.25, 1.14	0.10	0.54	0.21, 1.36	0.2
**Age (years)**	1.00	0.95, 1.05	0.97	1.00	0.94, 1.07	>0.9
**Histology**						

Mixed	—	—		—	—	
Urothelial	0.60	0.24, 1.48	0.27	0.39	0.13, 1.21	0.10
**Primary site**						
Bladder	—	—		—	—	
Upper GU	1.49	0.61, 3.67	0.38	2.47	0.85, 7.23	0.10
**Lymph node mets**						
Present	—	—		—	—	
Absent	0.98	0.46, 2.11	0.96	1.21	0.38, 3.91	0.7
**Lung mets**						
Present	—	—		—	—	
Absent	0.44	0.20, 0.94	0.035	0.51	0.17, 1.54	0.2
**Liver mets**						
Present	—	—		—	—	
Absent	0.65	0.26, 1.62	0.35	0.69	0.20, 2.44	0.6
**Bone mets**						
Present	—	—		—	—	
Absent	0.68	0.28, 1.61	0.38	0.54	0.18, 1.67	0.3
**Disease status**						
Locally advanced	—	—		—	—	
Metastatic	2.51	0.75, 8.35	0.13	1.82	0.33, 9.99	0.5

Abbreviations: CI = Confidence Interval, HR = Hazard Ratio

B. Model including treatment-emergent neuropathy

	Univariate			Multivariable		
**Characteristic**	**HR**	**95% CI**	**p-value**	**HR**	**95% CI**	**p-value**
**Neuropathy**						
Absent	—	—		—	—	

Present	0.32	0.14, 0.73	0.007	0.18	0.06, 0.52	0.002
**Age (years)**	1.00	0.95, 1.05	0.97	0.96	0.89, 1.03	0.2
**Histology**						
Mixed	—	—		—	—	
Urothelial	0.60	0.24, 1.48	0.27	0.41	0.13, 1.35	0.14
**Primary site**						
Bladder	—	—		—	—	
Upper GU	1.49	0.61, 3.67	0.38	2.42	0.87, 6.74	0.090
**Lymph node mets**						
Present	—	—		—	—	
Absent	0.98	0.46, 2.11	0.96	1.29	0.34, 4.83	0.7
**Lung mets**						
Present	—	—		—	—	
Absent	0.44	0.20, 0.94	0.035	0.44	0.13, 1.58	0.2
**Liver mets**						
Present	—	—		—	—	
Absent	0.65	0.26, 1.62	0.35	1.29	0.34, 4.85	0.7
**Bone mets**						
Present	—	—		—	—	
Absent	0.68	0.28, 1.61	0.38	0.33	0.10, 1.07	0.064
**Disease status**						
Locally advanced	—	—		—	—	
Metastatic	2.51	0.75, 8.35	0.13	2.03	0.34, 12.1	0.4

Abbreviations: CI = Confidence Interval, HR = Hazard Ratio

C. Model including EVP dose						
	Univariate			Multivariable		
**Characteristic**	**HR**	**95% CI**	**p-value**	**HR**	**95% CI**	**p-value**
Age (years)	1.00	0.95, 1.05	0.97	0.96	0.90, 1.04	0.3
EVP dose						
EV 1.0 mg/kg	—	—		—	—	
EV 1.25 mg/kg	0.30	0.13, 0.68	0.004	0.26	0.08, 0.86	0.027
Histology						
Mixed	—	—		—	—	
Urothelial	0.60	0.24, 1.48	0.27	0.54	0.17, 1.72	0.3
Primary site						
Bladder	—	—		—	—	
Upper GU	1.49	0.61, 3.67	0.38	1.57	0.50, 4.94	0.4
Lymphnode						
Metastatic	—	—		—	—	
Non-metastatic	0.98	0.46, 2.11	0.96	0.82	0.23, 2.89	0.8
Lung						
Metastatic	—	—		—	—	
Non-metastatic	0.44	0.20, 0.94	0.035	0.37	0.11, 1.26	0.11
Liver						
Metastatic	—	—		—	—	
Non-metastatic	0.65	0.26, 1.62	0.35	0.70	0.19, 2.54	0.6
Bone						
Metastatic	—	—		—	—	

Non-metastatic	0.68	0.28, 1.61	0.38	0.63	0.19, 2.05	0.4
Disease status						
Locally advanced	—	—		—	—	
Metastatic	2.51	0.75, 8.35	0.13	0.76	0.11, 5.19	0.8
Abbreviations: CI = Confidence Interval, HR = Hazard Ratio						

Supplementary Table S4. Univariate and multivariable Cox proportional hazards analyses for overall survival

**A. Model including treatment-emergent rash**						
	Univariate			Multivariable		
**Characteristic**	**HR**	**95% CI**	**p-value**	**HR**	**95% CI**	**p-value**
**Age (years)**	0.97	0.91, 1.03	0.35	1.00	0.92, 1.09	>0.9
**Rash**						
Absent	—	—		—	—	
Present	0.31	0.11, 0.91	0.032	0.26	0.07, 0.93	0.039
**Histology**						
Mixed	—	—		—	—	
Urothelial	0.32	0.11, 0.96	0.043	0.17	0.04, 0.70	0.014
**Primary site**						
Bladder	—	—		—	—	
Upper GU	1.55	0.47, 5.08	0.47	2.53	0.60, 10.6	0.2
**Lymph node mets**						
Present	—	—		—	—	
Absent	1.20	0.41, 3.49	0.74	0.59	0.11, 3.18	0.5
**Lung mets**						

Present	—	—		—	—	
Absent	0.37	0.13, 1.05	0.062	0.44	0.08, 2.43	0.3
**Liver mets**						
Present	—	—		—	—	
Absent	0.49	0.15, 1.56	0.23	0.25	0.05, 1.28	0.10
**Bone mets**						
Present	—	—		—	—	
Absent	0.60	0.19, 1.94	0.39	0.36	0.08, 1.52	0.2
**Disease status**						
Locally advanced	—	—		—	—	
Metastatic	1.54	0.34, 6.91	0.57	0.61	0.06, 6.33	0.7
Abbreviations: CI = Confidence Interval, HR = Hazard Ratio						

B. Model including treatment-emergent neuropathy

	Univariate			Multivariable		
**Characteristic**	**HR**	**95% CI**	**p-value**	**HR**	**95% CI**	**p-value**
**Age (years)**	0.97	0.91, 1.03	0.35	0.93	0.85, 1.03	0.2
**Neuropathy**						
Absent	—	—		—	—	
Present	0.24	0.07, 0.87	0.029	0.11	0.02, 0.54	0.007
**Histology**						
Mixed	—	—		—	—	
Urothelial	0.32	0.11, 0.96	0.043	0.13	0.03, 0.58	0.008
**Primary site**						
Bladder	—	—		—	—	
Upper GU	1.55	0.47, 5.08	0.47	2.41	0.56, 10.4	0.2

**Lymph node mets**						
Present	—	—		—	—	
Absent	1.20	0.41, 3.49	0.74	0.76	0.09, 6.60	0.8
**Lung mets**						
Present	—	—		—	—	
Absent	0.37	0.13, 1.05	0.062	0.49	0.06, 3.99	0.5
**Liver mets**						
Present	—	—		—	—	
Absent	0.49	0.15, 1.56	0.23	0.50	0.10, 2.60	0.4
**Bone mets**						
Present	—	—		—	—	
Absent	0.60	0.19, 1.94	0.39	0.21	0.04, 0.99	0.049
**Disease status**						
Locally advanced	—	—		—	—	
Metastatic	1.54	0.34, 6.91	0.57	0.63	0.04, 9.41	0.7
Abbreviations: CI = Confidence Interval, HR = Hazard Ratio						

C. Model including EVP dose

	Univariate			Multivariable		
**Characteristic**	**HR**	**95% CI**	**p-value**	**HR**	**95% CI**	**p-value**
Age (years)	0.97	0.91, 1.03	0.35	0.95	0.86, 1.05	0.3
EVP dose						
EV 1.0 mg/kg	—	—		—	—	
EV 1.25 mg/kg	0.32	0.10, 0.97	0.044	0.19	0.04, 0.94	0.042
Histology						

Mixed	—	—		—	—	
Urothelial	0.32	0.11, 0.96	0.043	0.23	0.05, 0.95	0.043
Primary site						
Bladder	—	—		—	—	
Upper GU	1.55	0.47, 5.08	0.47	1.78	0.41, 7.75	0.4
Lymphnode						
Metastatic	—	—		—	—	
Non-metastatic	1.20	0.41, 3.49	0.74	0.49	0.08, 2.92	0.4
Lung						
Metastatic	—	—		—	—	
Non-metastatic	0.37	0.13, 1.05	0.062	0.33	0.05, 2.16	0.2
Liver						
Metastatic	—	—		—	—	
Non-metastatic	0.49	0.15, 1.56	0.23	0.48	0.09, 2.65	0.4
Bone						
Metastatic	—	—		—	—	
Non-metastatic	0.60	0.19, 1.94	0.39	0.32	0.06, 1.62	0.2
Disease status						
Locally advanced	—	—		—	—	
Metastatic	1.54	0.34, 6.91	0.57	0.25	0.02, 3.46	0.3
Abbreviations: CI = Confidence Interval, HR = Hazard Ratio						

Supplementary Table S5: Landmark analyses of early rash and overall survival

Landmark	Patients included, n (rash/no rash)	Post-landmark deaths, n	HR (95% CI)	p-value
3 weeks	60 (23/37)	14	0.25 (0.06–1.13)	0.072
6 weeks	58 (26/32)	12	0.26 (0.06–1.18)	0.080

Supplementary Table S6: Treatment exposure[a] according to treatment-emergent rash and peripheral neuropathy

Toxicity group	n	EV cycles, median (IQR)	Pembrolizumab cycles, median (IQR)
**Rash present**	38	**8 (6–10.75)**	**9 (6.25–13.75)**
**Rash absent**	22	**5.5 (3.25–8)**	**5.5 (3.25–10.75)**
**Neuropathy present**	28	**8 (6–13)**	**12 (7–14.25)**
**Neuropathy absent**	32	**6 (3–8)**	**6.5 (3.75–9.25)**

[a]Treatment exposure represents the total number of EV or pembrolizumab cycles received during the treatment course, stratified by the presence or absence of treatment-related rash or neuropathy. Values are presented as median (IQR)

Supplementary Table S7. Characteristics, management, and treatment exposure among patients who developed treatment-emergent rash

Characteristic	Rash Cohort (n = 38)
**Rash characteristics**	
Time to first rash, days, median (IQR)	17 (11.5–57.3)
First rash within 3 weeks, n (%)	23 (60.5)
First rash within 6 weeks, n (%)	27 (71.1)
Grade 1–2 at first rash, n (%)	31 (81.6)
Grade 3–4 at first rash, n (%)	7 (18.4)
≥2 rash episodes, n (%)	15 (39.5)
**Rash-related management**	
EV dose reduction, n (%)	20 (52.6)
Treatment delay/hold, n (%)	14 (36.8)
Systemic corticosteroid use, n (%)	16 (42.1)
Dermatology consultation, n (%)	7 (18.4)
EV discontinuation due to rash, n (%)	4 (10.5)
Pembrolizumab discontinuation due to rash, n (%)	1 (2.6)
**Treatment exposure**	
EV treatment duration, days, median (IQR)	160.0 (119.3–261.5)
Pembrolizumab treatment duration, days, median (IQR)	196.5 (127.5–301.3)
